# Relationship between multiple drug resistance and biofilm formation in Staphylococcus aureus isolated from medical and non-medical personnel in Yaounde, Cameroon

**DOI:** 10.11604/pamj.2014.17.186.2363

**Published:** 2014-03-11

**Authors:** Agnes Bedie Eyoh, Michel Toukam, Julius Atashili, Charles Fokunang, Hortense Gonsu, Emilia Enjema Lyonga, Henshaw Mandi, George Ikomey, Bertha Mukwele, Martha Mesembe, Marie Claire Okomo Assoumou

**Affiliations:** 1 Faculty of Medicine and Biomedical Sciences, University of Yaounde 1, Cameroon; 2 Faculty of Health Sciences, University of Buea, Cameroon; 3 Ministry of Public Health, Yaounde, Cameroon; 4 Center for the Study and Control of Communicable Diseases, Cameroon

**Keywords:** Nosocomial, biofilm, multiple drug resistance, medical personnel, non-medical personnel

## Abstract

**Introduction:**

Monitoring the prevalence of nasal carriage of multiple drug resistance (MDR) *Staphylococcus aureus* (SA) strains in hospital personnel is essential. These strains when transmitted from hospital personnel to patients with already weakened immune states or in-built medical devices, may limit the latter's treatment options. This study aimed at assessing the potential exposure of patients to these MDR SA in a resource-limited hospital setting by assessing the prevalence and relationship between antimicrobial susceptibility and biofilm forming capacity of SA isolates from hospital personnel.

**Methods:**

A total of 59 bacteria isolates phenotypically identified as *Staphylococcus aureus* obtained from medical (39) and non-medical personnel (20) in Yaounde were used in the study. Multiple drug resistance defined as resistance to four or more of twelve locally used antibiotics were determined by Kirby Bauer disc diffusion technique whereas quantification of biofilm production was by the microtitre plate method.

**Results:**

Among the 59 SA isolates, the prevalence of MDR was 50.9%. Among medical personnel 48.7% had MDR as against 55.9% for non-medical personnel (p-value=0.648). The overall percentage of weak biofilm producers was 35.6%. Although the prevalence of weak biofilm formers was higher in isolates from non-medical personnel (40%) than medical personnel (33.3%) the difference was not statistically significant (p-value= 0.246). Slightly less than half (42.9%) of the weak biofilm producers were MDR.

**Conclusion:**

Considering the high rates of MDR and that slightly less than half of biofilm formers were MDR, these trends need to be monitored regularly among hospital personnel in Yaounde.

## Introduction

The development of multidrug resistance by *Staphylococcus aureus* (SA) especially methicilin resistant strains is a public health concern. This problem is further compounded in sub-Saharan Africa by the absence of systematic antibiotic susceptibility testing and thus the lack of appropriate guidelines for empiric treatment. In the hospital milieu, infected and colonized patients mediate the dissemination of *S. aureus* and hospital personnel, serving as reservoirs, ease further transmission [1, 2]. Factors such as poor hand hygiene have been associated with transmission rates of up to 40% [3]. Retrospective studies have shown an upsurge of methicillin resistance *staphylococcus aureus* (MRSA) which is also indicative of multiple drug resistance (MDR) [4–6]. Infections caused by MRSA often prove difficult to treat due to high levels of resistance to multiple antibiotics as a result of both intrinsic and acquired mechanisms [7].

Drug resistance in *S. aureus* is mediated by complex genetic arrays such as the staphylococcal chromosomal cassette mec elements for methicillin or the vanA operon acquired through horizontal gene transfer [8]. Other resistances against antibiotics like fluoroquinolones, linezolid and daptomycin have developed through spontaneous mutations and positive selection [9, 10]. Detection of the resistance pattern is therefore an important support tool to antibiotic treatment guidelines and susceptibility surveillance of *S. aureus* in areas where susceptibility testing is not a routine.

*S. aureus* biofilms have been associated with a variety of persistent infections which respond poorly to conventional antibiotic therapy [11]. Biofilm formations also help in the spread of antibiotic resistant traits in nosocomial pathogens by increasing mutation rates and by the exchange of genes which are responsible for antibiotic resistance [12]. Biofilm formation is regulated by expression of polysaccharide intracellular adhesion antigens (PIA), which mediates cell-to-cell adhesion and is the gene product of *ica* ADBC [13]. Many biofilm infections develop gradually, producing very few symptoms initially, but in the long run, may produce immune complex sequelae and may also act as reservoirs of infection through sloughing [11]. Biofilm-producing *S. aureus* is known to be more difficult to control, having greater resistance to antibacterial agents than *S. aureus* not embedded in biofilm. Biofilm-producing strains consequently, when transported to sterile body sites of carrier, transmitted to patients with already weaken immune states or with inbuilt medical devices, may complicate treatment options especially in resource limited settings were assays for the detection of biofilm production are not readily available. Besides, standard in vitro antibiotic susceptibility tests are not done routinely and also not predictive of the therapeutic outcome of biofilm associated infections.

A better understanding of the associations between MDR patterns, biofilm production and transporter state of SA among hospital personnel could provide data for guidelines to better manage *S. aureus* infections of nosocomial origin in resource-limited settings.

## Methods

In this study, we analyzed 59 SA strains isolated from medical and non-medical personnel of three health institutions in Yaounde, Cameroon. Medical personnel in this study were defined as personnel with direct patient contact and non-medical as personnel without direct patient contact. These strains were phenotypically identified as *S. aureus* based on growth and fermentation of mannitol salt agar, colonial morphology on nutrient agar, characteristics upon Gram staining and coagulase tests, plus the presence of DNA (DNase test), protein A and clumping factor (SLIDEX^®^ Staph plus, BioMerieux, Marcy -I'Etoile, France), and biochemical properties (API Staph identification System,BioMerieux, Marcy -I'Etoile, France).

### Biofilm assay

Quantification of Biofilm production was performed using the microtitre plate method [14]. This assay was performed using U-shaped polystyrene microtitre plates, with each well containing 199 µl of Brain Heart Infusion broth (BHI) supplemented with 1% sucrose. Into each of these wells was added 1µl of S. aureus isolates grown in BHI for 3 hours. Each isolate was run in triplicate. The plates were then covered with cover seals and incubated at 37°C for 24 h. After incubation, plates were emptied and washed five times with Phosphate Buffered Saline (PBS). Then, 175 µl of 1% crystal violet was added and incubated at room temperature for 15 min. The plates were further washed 5 times with PBS and dried for 30 min at room temperature. Two hundred µl of ethanol-acetone (80% / 20%) was added and plates incubated at room temperature for 25 min. Wells with sterile BHI alone were used as controls. *S. aureus* 25923 was used as the positive control. Absorbance of the adherent cells was measured at 490 nm using a (Mindray 96-MR) microplate reader. A strain was considered a non-producer, if its absorbance value was <0.12(optical density). Those with optical density values between 0.120 and 0.240 were considered moderate or weak producers and those with optical density values more than 0.240 were considered strong producers.

### Multiple drug resistance

Multiple drug resistance (MDR) was defined in this study as resistance of an isolate to 4 or more of the twelve tested antibiotics. MDR was determined by the Kirby Bauer disc diffusion method. An ATCC 25923 control strain was included in the tested isolates. Inhibition zone diameters for each antimicrobial was measured and interpreted as outlined by Clinical Laboratory Standard Institute (CLSI, 2007) [15].

Ethical clearance for this study was obtained from the ethical committee of the Faculty of Medicine and Biomedical Sciences of the University of Yaounde I.

### Statistical analysis

Data collected were entered into a spreadsheet and analyzed using STATA (STATA corps, Texas, USA). Proportions were compared using Chi-Square tests or Fisher's exact tests, as appropriate. The levels of statistical significance was set at a p-value < 0.05

## Results

### Multiple drug resistance and personnel types

The SA strains used in the study were isolated from medical and non-medical personnel (Table 1). The antimicrobial susceptibility test results are shown in Table 2. Isolates from both groups were generally highly resistant to penicillin, doxycycline and erythromycin. They have generally very low resistance to ampicillin, cephalotin, pristimycin. There was however no significant difference in the resistance pattern between the two groups. The overall prevalence of MDR was 50.9%. However, as indicated in Table 3, MDR was more prevalent among non-medical personnel (55.9%) than medical personnel (48.7%) The difference was however not statistically significant (p=0.648)

**Table 1:** Frequency and percentage distribution of isolates according to sex and personnel type

Personnel Type	Male (%)	Female (%)	Both groups (%)
**Medical**	11(47.8)	28(77.8)	39(100)
**Non-medical**	12(52.2)	8(22.2)	20(100)
**Total**	**23(100)**	**36(100)**	**59(100)**

**Table 2:** Comparison of resistance pattern of *S. aureus* between medical personnel and non-medical personnel in Yaounde, Cameroon

Antibiotic (potency)	Isolates resistant to antibiotic		
	MP	NMP	
	N=39	N=20	
	No. (%)	No. (%)	p-value*
Amikacin(30µg)	3(7.7)	0(0.0)	0.54
Cephalotin (30µg)	0(0.0)	1 (5.0)	0.34
Chloramphenicol(30µg)	1(2.6)	1(5.0)	1.00
Ciprofloxacin (5µg)	5(12.8)	1(5.0)	0.65
Doxycycline(30µg)	13 (33.3)	5(25.0)	0.79
Erythromycin (15µg)	22(56.4)	13(65.0)	0.69
Gentamicin(10µg)	5 (12.8)	1(5.0)	0.77
Netilmicin	0(0)	0(0.00)	1.00
Oxacillin(0.016-256µg)	5 (13.1)	3(15.0)	1.00
Penicillin (6µg)	39(100)	20(100)	1.00
Pristimycin(15µg)	2(5.6)	1(5)	1.00
Vancomycin (10µg)	0 (0)	0(0.00)	1.00

Number of resistant isolates, MP. Medical Personnel, NMP. Non-Medical personnel

* Fisher's exact p-value

**Table 3:** Relationship between multiple drug resistance and personnel type

	**Multiple drug resistance**			
**Personnel**	**<4 (%)**	**>4(%)**	**Total (%)**	**p-value**
Medical	20(51.3)	19(48.7)	39	
Non-Medical	9(44.1)	11(55.9)	20	0.648
**Total**	**29(49.1)**	**30(50.9)**	**59**	

<4 resistant to less than four antibiotics: >4 resistant to more than 4 antibiotics; p-value from a Pearson Chi Square test.

### Biofilm formation and personnel type

Out of the 59 isolates tested for biofilm production, 21(35.6%) were identified as biofilm formers. These biofilm formers all had OD values between 0.120 and 0.240 thus were considered as moderate or weak biofilm producers. Amongst the 21 biofilm producing isolates 13(33.3%) were from medical personnel and 8(40.0%) from non-medical personnel (Table 4).

**Table 4:** Relationship between biofilm production and personnel type

	**Biofilm**			
**Personnel**	**Non producer**	**Weak Producer**	**Total**	**p-value**
Medical	26(66.7%)	13(33.3%)	39	
Non-Medical	12(60.0%)	8(40.0%)	20	0.246
**Total**	**38(64.4%)**	**21(35.6%)**	**59**	

P-value from a Pearson Chi Square test

### Biofilm formation and multiple drug resistance

MDR was detected in 9 (42.9%) of biofilm forming isolates, with 4(50%) from non-medical personnel and 5(38%) from medical personnel. There were no significant differences in the percentage of MDR among weak biofilm producers and non- biofilm producing strains for both medical (p= 0.37) and non-medical personnel (p= 0.71) (Table 5).

**Table 5:** Relationship between multiple drug resistance and biofilm production by personnel type

	Medical personnel (N=39)			Non-Medical personnel (N=20)		
	n	MDR (%)	p-value	n	MDR (%)	p-value
**Non Biofilm**	26	14 (54)	0.37	12	7 (58)	0.71
**Weak Biofilm**	13	5 ( 38)		8	4 (50)	
**Total**	39	19 (49)		20	11 (55)	

P-value from a Pearson Chi Square test

## Discussion

This study describes and compares multiple drug resistance patterns and biofilm production of *S aureus* strains isolated from medical (39) and non-medical personnel (20) in Yaounde, Cameroon. Over 50% of the isolates from both groups were found to be resistant to more than four of the antibiotics employed in the study. While no resistance was recorded for vancomycin and netilmicin, high levels were recorded for penicillin, erythromycin and doxycycline in both groups. Biofilm production was identified in 21(35.6%) of the isolates with non-medical personnel registering a higher prevalence.

Several studies have implicated hospital personnel in the transmission of SA within nosocomial context. In addition, they are known to harbor higher rates of MDR resistant strains in the anterior nares than the general population [16]. The total prevalence of MDR in the present study was 50.9%. In similar studies, 69% was recorded for health care workers in a Yemen hospital, 21.95% in Pakistan and 13.6% among hospital personnel in the US. Among the medical personnel 48.7% had MDR as against 55.0% for non-medical personnel with the computed p-value of < 0.648. Our findings were contrary to the study that found out that medical personnel were colonized with more antibiotic-resistant strains than nonmedical personnel (mean, 2.8 versus 2.1 isolates < 0.03’ > P < 0.03) [17]. These contradictory findings could be explained by the difference in settings with different local lifestyle and poor hygiene condition. Biofilm is one of the important microbial virulence factors found in S. aureus. Bacteria use biofilm mechanism as a way of causing chronic infection to human [17–19]. Biofilm are also well suited for resistance to antibiotics and evasion of immune system's defenses. Furthermore, biofilm-mediated infections in the hospital environment with hospital personnel as a steady reservoir has a significant negative impact on patient?s health and places an enormous burden on the financial resources of the individual [20]. The study recorded most biofilm producers (weak) from non-medical personnel with 40% as compared to 33.3% for medical personnel. The ability of *S. aureus*, to form biofilms is of significant clinical importance, since biofilm formation influences the effectiveness of antimicrobial therapy, the subsequent outcome of an infection, increased prevalence of antibiotic resistance and induce resistance also to vancomycin [19]. However, weak producers identified among non-medical personnel were significantly resistant to erythromycin (p= 0.01).

There was no significant difference in the percentage of MDR among biofilm producers and non- biofilm formers for both medical and non-medical personnel. Some other studies have found more MDR stains among biofilm producers than non- biofilm formers [21]. MDR in biofilm forming SA has been partly attributed to the extracellular polymeric substances constituting this matrix serving as a diffusional barrier for antibiotics, thus influencing either the rate of transport of the molecule to the biofilm interior or the reaction of the antimicrobial material with the matrix material [22].

Our study had some limitations. We did not evaluate the impact of nasal carriage of MDR/Biofilm forming *S. aureus* on transmission and nosocomial infection in patients receiving healthcare in this setting. Secondly, drug resistance was analyzed phenotypically and not genetically. We therefore recommend further studies involving a much larger number of isolates, and molecular identification of genes responsible for resistance and biofilm formation.

## Conclusion

Although there was no significant difference in the prevalence of MDR and Biofilm formation between medical and non-medical personnel, both groups can be sources of highly pathogenic strains of SA. Thus both groups ought to be targeted in any interventions aimed at reducing hospital-acquired SA.
